# Developing an efficient intercrop system for understory medicinal plant cultivation: allelopathy- based screening of tree species for *Turpinia arguta*

**DOI:** 10.3389/fpls.2026.1878126

**Published:** 2026-07-03

**Authors:** Shengchun Li, Shaoyong Deng, Xiaorui Huang, Xiaoli Yang, Xiongxiong Xie, Xiaohong Hu, Fengying Qiu, Songsong Zhou, Haikuan Yang, Junhuo Cai, Xiuhua Tao

**Affiliations:** 1 Jiangxi Academy of Forestry, Nanchang Jiangxi, China; 2 College of Landscape and Art, Jiangxi Agricultural University, Nanchang, China

**Keywords:** allelopathy, medicinal plant, *Pinus elliottii*, rhizosphere soil aqueous extracts, *Turpinia arguta*, understory cultivation

## Abstract

Allelopathy is a common regulatory interaction among plants in nature, understanding these allelopathic interactions offers a promising bio-tool for guiding or designing productive and sustainable agroforestry systems. However, due to species-specific differences, how to quantitatively and systematically select appropriate nurse trees based on allelopathic effects to optimize understory cultivation of medicinal plants remains largely unclear. This study evaluated the potential of utilizing allelopathic effects of rhizosphere soil aqueous extracts to select nurse trees for establishing efficient understory cultivation system for *Turpinia arguta*, a valuable shade-tolerant medicinal plant with substantial market demand. We tested the allelopathic effects of rhizosphere soil aqueous extracts from seven candidate companion tree species of *T. arguta* on its seed germination, seedling growth and cutting physiology. Results revealed a concentration-dependent allelopathic response: seed germination was optimally promoted at 0.25 g/mL of the aqueous extracts of *Pinus elliottii* and *Camphora officinarum*. Notably, the aqueous extract of *P. elliottii* consistently exhibited the greatest growth-promoting effects, significantly enhancing seedling root length, plant height and biomass (fresh and dry weight). For cuttings, the aqueous extracts generally promoted Pn and Tr, while low-concentration of aqueous extracts from *P. elliottii*, *C. officinarum* and *Liquidambar formosana* increased the actual photochemistry efficiency of PSII (YII), qP and ETR. Furthermore, MDA content, SOD, POD and CAT were dependent on both tree species and the concentrations of aqueous extracts. The synthetic allelopathic index confirmed *P. elliottii* as the most beneficial companion tree for *T. arguta*. Taken together, this study shows that *P. elliottii* has consistently positive allelopathic effects on *T. arguta*, promoting its seed germination and seedling growth, and these findings support an integrated *P. elliottii*- *T. arguta* understory planting system. Similar allelopathic evaluation approaches can be used to select optimal companion trees for other valuable medicinal plants in diversified agroforestry systems.

## 1 Introduction

*Turpinia arguta* (Lindl.) Seem. is an evergreen shrub belonging to the family Staphyleaceae, which is primarily distributed in Jiangxi, Hunan, Guangxi, Guizhou and Fujian provinces of China. This species is recognized for its high pharmacological value, particularly in treating pharyngitis, tonsillitis, and tonsillar abscesses (Yang et al., 2021). *T*. *arguta* was officially documented in *Pharmacopoeia of the People’s Republic of China* (Ch.P.) in 2010. Its dry leaves, termed as *Turpiniae folium* in the Ch.P., contains various chemical constituents, including flavonoids, triterpenoids and phenolic acids, with ligustroflavone and rhoifolin being the most effect medicinal components (Xiao et al., 2019; National Pharmacopoeia Committee, 2020). *Turpiniae folium* possesses versatile pharmacological functions, such as anti-inflammatory, antipyretic and antidotal effect, anti-bacterial and analgesic effect, as well as the ability to promote blood circulation (National Pharmacopoeia Committee, 2020; Liu et al., 2022).

In recent years, natural products from plants have become imperative sources of medicinal agents, phytomedicine has gained increasing interest as new concept for health management (Azab et al., 2016; Pu et al., 2017; Wang et al., 2017; Zhang et al., 2017; Wang et al., 2018). Traditional Chinese medicine resources are the cornerstone of the high-quality development of the whole traditional Chinese medicine industry chain, and authentic Chinese medicine is the benchmark of Chinese medicine market (Xin et al., 2019). With the continuous improvement in living standards, public awareness of health care has grown considerably. Notably, influenced by the COVID-19 epidemic, the demand for traditional Chinese medicine has significantly increased. *T*. *arguta* is a national protected variety of traditional Chinese medicine, the demand for products which using it as raw materials has been increasing recently (Xu, 2011; Wu, 2014), and *T*. *arguta* has emerged as a high-value industrial crop. In the past, the supply of *T*. *arguta* primarily relied on wild resources. With the continuous reduction of wild resources and the increasing demand for *T*. *arguta*, the supply of raw materials for *T*. *arguta* has been insufficient. In order to meet market requirements, artificial cultivation has become a preferred way. Under-forest economy is a new direction of modern planting and supported by the Chinese government, as making full use of forestland to open up new planting channels. It not only solves environmental problems, but also increases farmers’ income. Forest-medicine planting patterns should be applied following the principle of ecological priority, and it is critically important to choose the suitable combination mode of tree species and medicine plants considering the allelopathy between plants.

As a small shade-tolerant shrub, *T*. *arguta* is naturally suited to understory systems. However, choosing the right type of trees can be challenging because of allelopathy between plants. Allelopathy is an ecological phenomenon which defined as an inhibitory or beneficial chemical interference between plants (Einhellig, 1995). Allelochemicals must be produced by plants and released into the environment to affect the survival, growth, development and reproduction of other plants (Hierro and Callaway, 2021). Allelochemicals can be classified into simple unsaturated lactones, simple phenols, benzoic acid and its derivatives, water-soluble organic acids, straight-chain alcohols, aliphatic aldehydes, ketones, long-chain fatty acids, polyacetylenes, benzoquinone, anthraquinone and complex quinones, flavonoids, terpenoids and steroids, coumarin, cinnamic acid and its derivatives, amino acids and peptides, alkaloids and cyanohydrins, purines and nucleosides, sulfide and glucosinolates and tannins (Rice, 1984). Allelochemicals are released from roots, stems, leaves, flowers and seeds of plants, and the release of allelochemicals to the environment is mainly through root exudates, leaching, volatilization, and decomposition of surface litters (Koocheki et al., 2013). These compounds can modulate the permeability of plant cell membrane, photosynthesis, respiration, enzyme activity and hormone level (Hejl et al., 1993; Baziramakenga et al., 1995; Penuelas et al., 1996). In addition, allelopathy could also affect plant cell division, elongation and submicroscopic structure, water absorption and ion transport in plants, gene expression and protein synthesis, as well as soil microbial environment (Cheng and Cheng, 2015; Kumar et al., 2024). In general, allelopathy effect can be evaluated through seed germination, seedling growth, and physiology, such as photosynthetic rate, physiological and biochemical indices of leaves (Wang et al., 2019; Li et al., 2021a; Hammami et al., 2024; Wang et al., 2024). However, most previous studies have focused on either a single indicator (e.g., seed germination) or a single tree-crop interaction, or have used leaf litter extracts rather than rhizosphere soil extracts-the latter being more representative of field conditions.

In the wild, *T*. *arguta* often grows under *Pinus elliottii* Engelm., *Camphora officinarum* Nees, *Liquidambar formosana* Hance, *Paulownia fortune* (Seem.) Hemsl., *Phoebe zhennan* S.K.Lee & F.N.Wei, *Choerospondias axillaris* (Roxb.) B.L.Burtt & A.W.Hill and *Cunninghamia lanceolata* (Lamb.) Hook., and these seven species are the main economic trees in southern China with extensive cultivation area, making them highly suitable for forest-medicine intercropping systems. Previous studies have shown that the interplanting patterns under forests of *Pa*. *fortune*, *Ph*. *zhennan*, *Ch*. *axillaris and Cu*. *lanceolata* can promote the growth of crops and medicinal plants (Tian et al., 2013; Pan et al., 2022; Yu, 2023; Wu et al., 2024). The rhizosphere soil of trees has significant allelopathic effects on the growth of adjacent plants (Duan et al., 2015b; Wang et al., 2019), and Duan et al. (2015a) reported that the rhizosphere soil aqueous extract includes more than 60 Allelochemicals. While some of these trees are already used in agroforestry, their allelopathic effects on *T*. *arguta* have not been quantitatively or systematically evaluated.

In order to explore the optimal forest type for the artificial cultivation of *T*. *arguta*, in this study, we tested the allelopathic effects of rhizosphere soil aqueous extracts from seven associated tree species of *T. arguta* on its seed germination, seedling growth and cutting physiology. Our objective is to identify the most compatible tree species for establishing a productive *T. arguta* understory cultivation system. This study aims to provide a scientific basis for optimizing species selection in agroforestry, thereby supporting the development of a reliable raw material supply chain for the *T. arguta* industry.

## 2 Methods

### 2.1 Preparation of rhizosphere soil aqueous extracts

The rhizosphere soil of *P*. *elliottii*, *C*. *officinarum*, *L*. *formosana*, *Pa*. *fortune*, *Ph*. *zhennan*, *Ch*. *axillaris* and *Cu*. *lanceolata* were collected from the same forest plot in Jiangxi Academy of Forestry. 500g rhizosphere soil was extracted in 500 ml distilled water for 12 hours for each species, during which the samples were placed on rotary shaker (150r/min), and then centrifuged for 15 min at 4000 rpm. The undiluted extracts with a mass concentration of 1 g/mL were obtained by filtration, and refrigerated at 4 °C as a stock. The undiluted extracts were aliquoted into 0.1 g/mL, 0.25 g/mL, 0.5 g/mL concentration gradients for biological tests.

### 2.2 Seed germination experiment

The experiment was conducted in a greenhouse at Jiangxi Academy of Forestry (115°82’ E, 28°74’ N). Seeds of *T*. *arguta* were collected from Jiulian Mountain, Ganzhou, Jiangxi province. Seeds of *T*. *arguta* were soaked in distilled water for 24 hours before sowing, then sterilized with 5% NaClO for 10 min and rinsed with distilled water for 5 times to avoid pathogen contamination. On March 29, 2023, three seeds were sown per container bag and covered with approximately 2 cm of substrate and 9 mL of different concentrations (0.1 g/mL, 0.25 g/mL, 0.5 g/mL) of the corresponding aqueous extract was added to each treatment. Each treatments comprised 30 seeds, and distilled water was used as the control. A total of 21 treatments were set up with three replications. Germination was monitored daily, and an appropriate amount of the respective aqueous extract was replenished. On the 41^st^ day, the test was completed and the germination rate (GR) and germination index (GI) were calculated. The germination potential (GP) was calculated on the 31^st^ day. In addition, seedling height, the longest root length, fresh weight and dry weight were measured. The indices were calculated according to the following equations (Kumar et al., 2011; Moori and Ahmadi-Lahijani, 2020; Saffari et al., 2021):


$$\text{Germination rate}\left(\text{GR}\right)=\frac{G_{f}}{N}\times 100\%$$

$$\text{Germination potential}\left(\text{GP}\right)=\frac{G_{s}}{N}\times 100\%$$

$$\text{Germination index}\left(\text{GI}\right)=\sum \frac{Gt}{Dt}$$

G_f_: the total number of germinated seeds at the end of the experiment.

N: the total number of seeds tested.

G_s_: the number of germinated seeds within 31days.

Dt: the number of germination days.

Gt: the number of germinated seeds per day corresponding to Dt.

### 2.3 Pot experiment

The cuttings of *T. arguta* were used for pot experiment. The cuttings of *T. arguta* were collected from Yongxin, Ji’an City. The experiment was conducted in the glass greenhouse of Jiangxi Academy of Forestry. Cuttings of uniform size were transplanted into plastic pots (D = 20 cm, H = 18 cm) on June 3, 2023, the substrate consisted of yellow core soil: perlite: peat soil: organic fertilizer at a ratio of 9:3:3:1. Each pot contained three cuttings, and each pot was considered as one experimental unit (the three cuttings within a pot were averaged for analysis). The cuttings were acclimatized for 30 days before the allelopathy experiment. The cuttings were irrigated weekly with 210 mL of the corresponding aqueous extract treatment, with distilled water serving as the control group. Each treatment had three replicates. The pot experiment was conducted for 90 days. Ninety days later, the photosynthetic indices, chlorophyll fluorescence parameters and physiological and biochemical indices were investigated.

### 2.4 LC-MS analysis

1 mL aqueous extract was mixed with 2 mL methanol acetonitrile solution (1:1, v/v). The mixture was vortexed for 60s, low-temperature ultrasonic for 30 mins, followed by centrifugation at 12000 rpm at 4 °C for 10 mins to obtain supernatant, and the supernatant was placed at -20 °C for 1 hour to precipitate proteins. A second centrifugation was performed at 12000 rpm at 4 °C for 10 mins. The obtained supernatant was lyophilized and reconstituted in 100 ul of 30% ACN, vortexed and centrifuged at 12000 rpm at 4 °C for 10 mins, and then the supernatant was taken for instrumental analysis. The original data was processed using the metabolomics software Progenesis QI (Waters Corporation, Milford USA).

### 2.5 Photosynthetic parameters, physiological and biochemical characteristics analysis

Photosynthetic parameters were measured using CIRAS-3 portable photosynthesis system (PP Systems USA). The leaf photosynthetic parameters of the cuttings were measured from 9:00 to 12:00 am in sunny weather. For each plant, three mature and healthy leaves were selected, with three measurements per leaf, and each treatment was repeated three times. The measured parameters included: stomatal conductance (Gs), Net photosynthetic rate (Pn), Intercellular CO_2_ concentration (Ci). The chlorophyll fluorescence parameters of leaves were measured by WALZ PAM-2500 portable modulated chlorophyll fluorescence analyzer (German). Before the measurement, the leaves were dark-adapted for 15–20 mins. The measured parameters included: Initial fluorescence (Fo), Maximum fluorescence (Fm), Actual photochemical efficiency of PSII (YII), Electron transport rate (ETR), Photochemical quenching coefficient (qP) and Maximum photochemical efficiency of PSII. Malondialdehyde content (MDA), SOD activity, POD activity and CAT activity in leaves were measured by spectrophotometer.

### 2.6 Synthetical allelopathic effect index

Allelopathic response index (RI) was calculated according to Williamson and Richardson (1988). The indices were calculated according to the following equations:


$$\text{RI}=1-\frac{\text{C}}{\text{T}}\left(\text{T}\geq \text{C}\right)$$

$$\text{RI}=\frac{\text{T}}{\text{C}}-1\left(\text{T}<\text{C}\right)$$

T: treatment, C: Control. When RI > 0, there is a promotion effect; When RI< 0, there is an inhibitory effect.

The synthetical allelopathic effect (SE) was the arithmetic average of the response indices of RI values under the same treatment.

### 2.7 Statistical analysis

The statistical analysis was processed with SPSS 26.0. All data were expressed as the mean ± standard deviation (SD). A two-way ANOVA was performed to analyze significant differences in the effects of tree species and different concentrations of rhizosphere soil extracts on seed germination, seedling growth and cutting physiology. The significant level was p<0.05. Origin 2018 was used to plot the data.

## 3 Results

### 3.1 Identification of chemical components in rhizosphere soil aqueous extracts

A total of 9 types of organic compounds were identified in aqueous extracts from seven tree species, which belonged to amides, terpenes, alcohols, glycosides, phenols, ketones, esters, acids and phenylpropanoids (**Supplementary Tables 1**–**S7**). Amides showed the highest content in aqueous extracts of *P. elliottii*, *L. formosana*, *Pa. fortune*, *Ph. zhennan*, *Ch. axillaris* and *Cu. lanceolata*, and phenols showed the highest content in aqueous extracts of *C. officinarum*. In addition, the glycosides were unique to *P. elliottii* and *C. officinarum*.

### 3.2 Effect of rhizosphere soil aqueous extracts on seed germination

**Figure 1:**
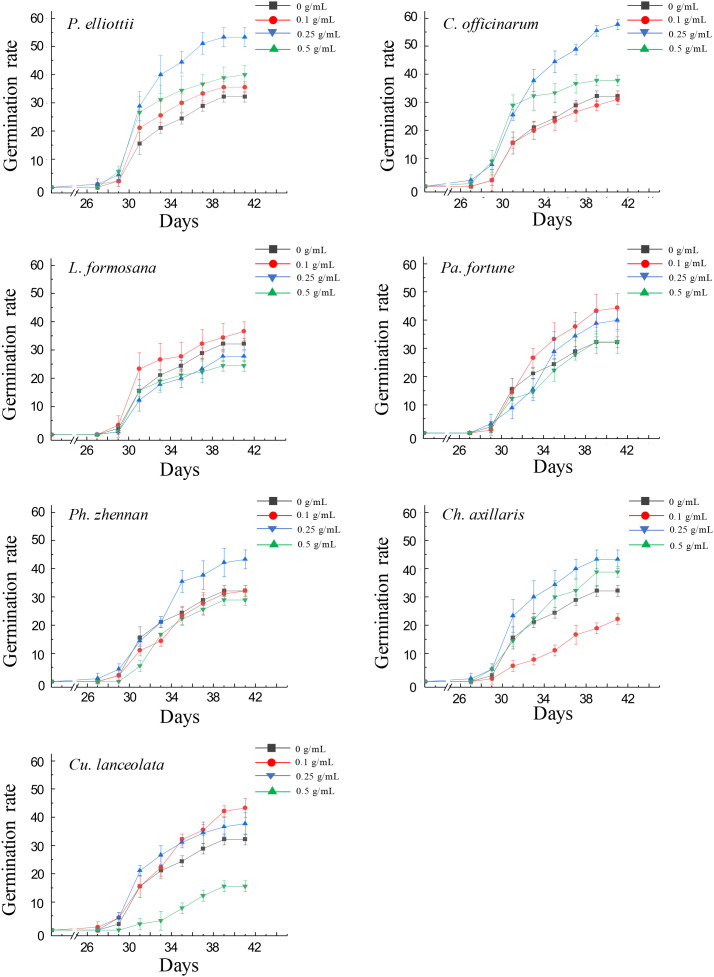
Effects of different concentrations of rhizosphere soil aqueous extracts from seven trees on germination rate of *T. arguta* seeds over time.

**Figure 2:**
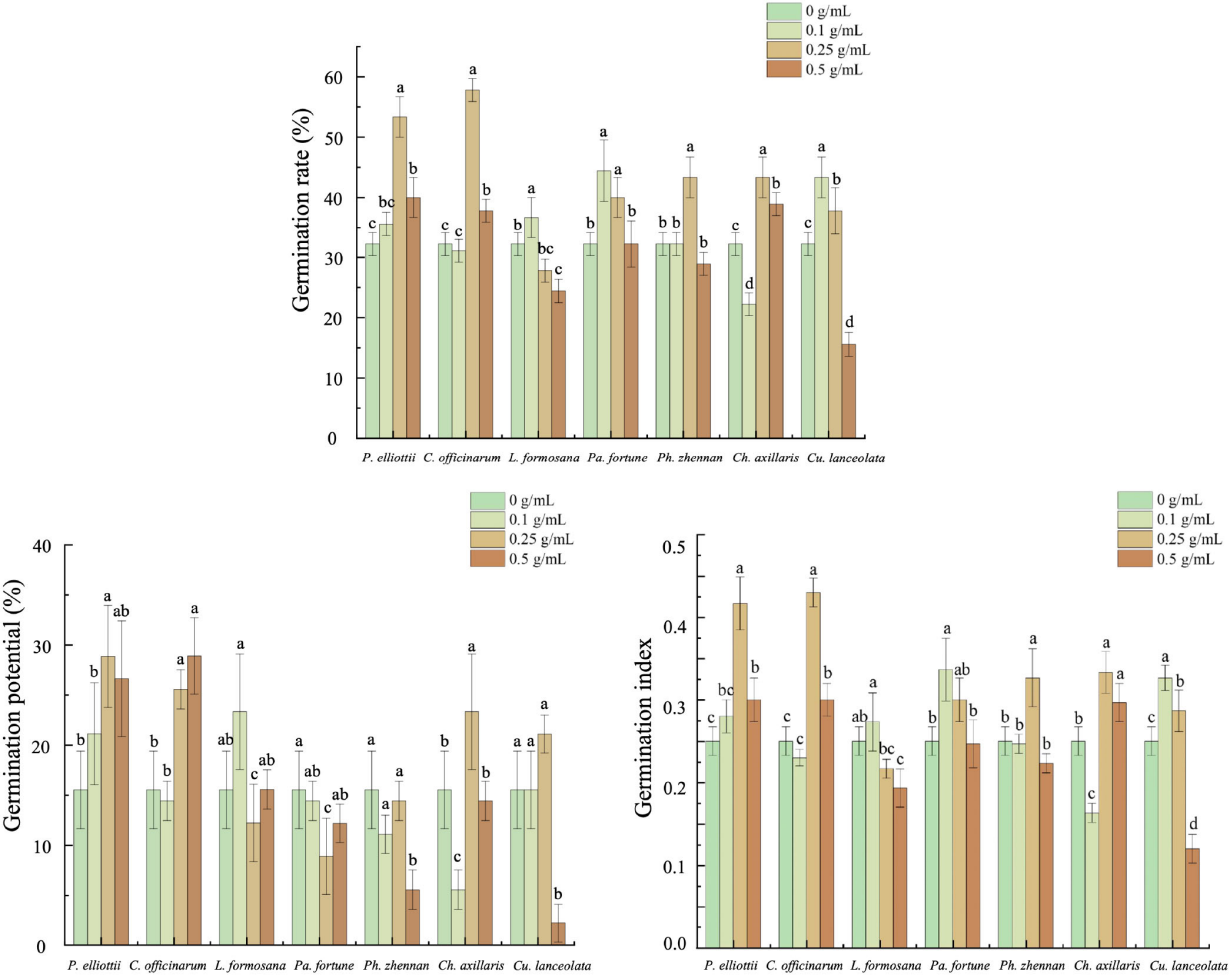
Effect of different concentrations of the rhizosphere soil aqueous extracts from seven trees on the germination rate, germination potential and germination index of *T. arguta* seeds. Lowercase letters represent significant differences in each treatment group (p< 0.05).

The seed germination rate of *T. arguta* increased gradually over time under the treatments of rhizosphere soil aqueous extracts from seven tree species (**Figure 1**). The highest number of germinations was concentrated between 29–31 days, and all germinations test were completed within 39–41 days. The seed germination rate exhibited a clear concentration-dependent trend response to the rhizosphere soil aqueous extracts (**Figures 1**, **2**). The control group showed a baseline germination rate of 32.2%. The rhizosphere soil aqueous extracts from *P*. *elliottii* exhibited a promotive effect on the seed germination rate, with the maximum promotional effect observed at the concentration of 0.25 g/mL, where the germination rate reached 53.3%- 1.6 times higher than that of the control group. Similarly, The greatest promotion effect (57.8%) on seed germination was also observed at the concentration of 0.25 g/mL for *C. officinarum*. The seed germination rate exhibited a low-concentration promotion and high-concentration inhibition trend under the treatment of aqueous extract from *L. formosana*, and the optimal promotion effect (36.67%) was observed at a concentration of 0.1 g/mL. When the concentration increased to 0.25 g/mL, the germination rate (27.8%) was lower than that of the control group, indicating an inhibitory effect. The rhizosphere soil aqueous extract from *Pa*. *fortune* exhibited a promotive effect on the seed germination rate at concentration of 0.1 g/mL and 0.25 g/mL. The greatest promotion effect (43.3%) on seed germination was under the treatment of aqueous extract from *Ph*. *zhennan* at the concentration of 0.25 g/mL, while other concentrations had no significant effects on the germination rate. Low-concentration (0.1 g/mL) aqueous extract from *Ch*. *axillaris* had an inhibitory effect (22.2%) on seed germination rate, but positive effects was observed at the concentration of 0.25 g/mL (43.3%) and 0.5 g/mL (38.9%). *Cu*. *lanceolata* exhibited a similar trend with *Pa*. *fortune* (**Figure 1**).

The control group showed a germination potential of 15.53% and germination index of 0.25. The greatest promotion effect on seed germination potential and germination index were observed under the treatment of aqueous extracts from *P. elliottii* (28.87%, 0.42) and *C. officinarum* (28.9%, 0.43) (**Figure 2**). The aqueous extracts from *Pa. fortune* and *Ph. zhennan* had an inhibitory effect on seed germination potential. The seed germination potential and germination index exhibited a low-concentration promotion and high-concentration inhibition trend under the treatment of aqueous extract from *L. formosana*. Low-concentration (0.1 g/mL) aqueous extract from *Ch. axillaris* had the strongest inhibitory effect on seed germination potential and germination index, while *Cu. lanceolata* showed the greatest inhibitory effect at concentration of 0.5 g/mL (**Figure 2**).

### 3.3 Effect of rhizosphere soil aqueous extracts on seedlings

**Figure 3:**
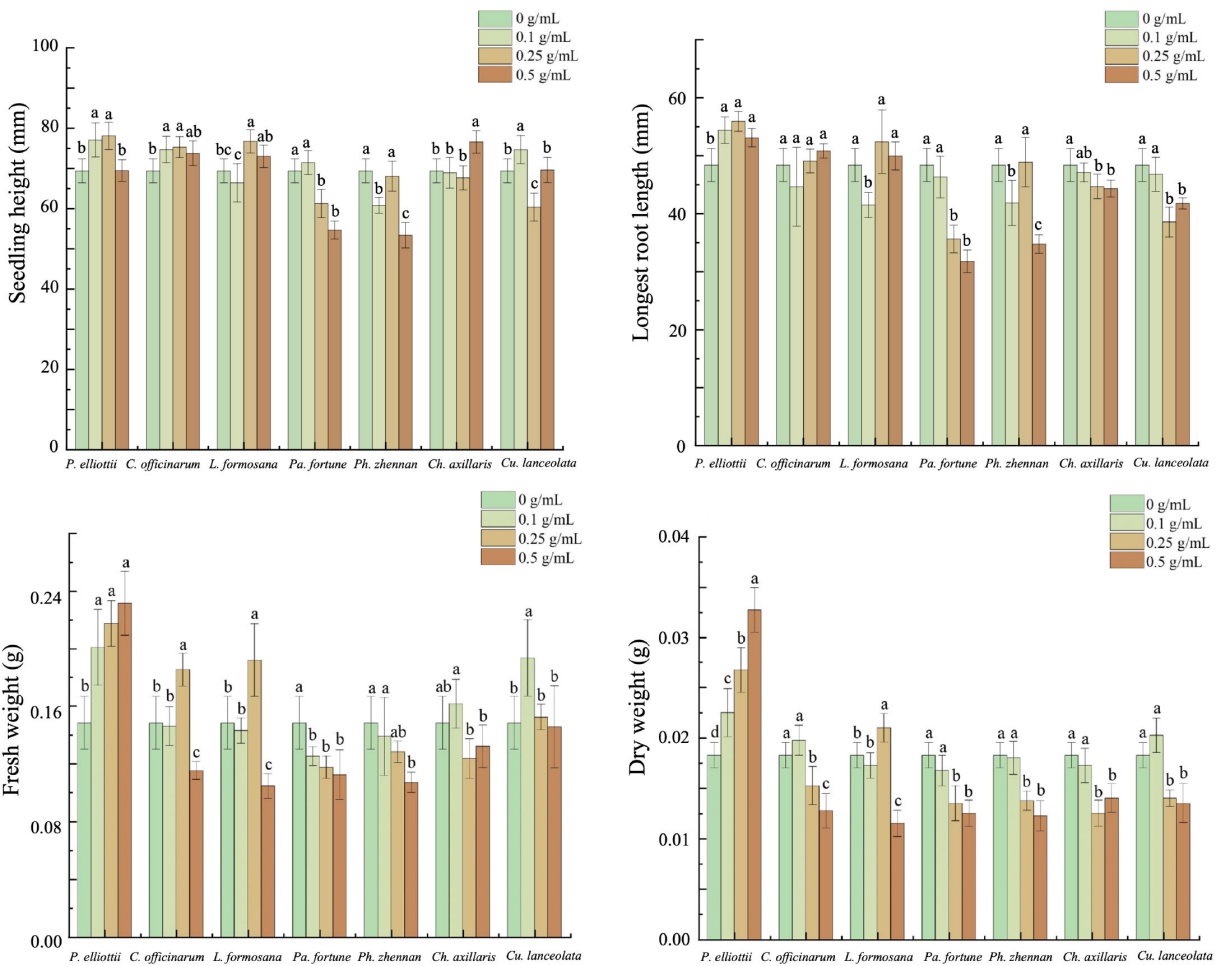
Effect of different concentrations of the rhizosphere soil aqueous extracts from seven trees on the seedling height, longest root length, fresh weight and dry weight of *T. arguta* seeds. Lowercase letters represent significant differences in each treatment group (p< 0.05).

The seedling height of control group was 69.4 mm. The aqueous extract from *P. elliottii* exhibited maximal growth-promoting on seedling height, the optimal promotion was observed at the concentration of 0.25 g/mL, and the height reached 78.12 mm (**Figure 3**). *C. officinarum* had significant promoting effects on seedling height, while *Ph. zhennan* had an inhibitory effect. The seedling height exhibited a low-concentration inhibition and high-concentration promotion trend under the treatment of aqueous extract from *L. formosana* and *Ch. axillaris*, but opposite patterns was observed from *Pa. fortune* and *Cu. lanceolata*. For the longest root length, only *P. elliottii* had a significant promoting effect (**Figure 3**).

The aqueous extracts from *P. elliottii* exhibited remarkable promoting effects on both fresh and dry weight of seedlings, and the maximum promotive effects on fresh and dry weight were 1.6 and 1.8 times than that of the control group (**Figure 3**). The aqueous extracts from *C. officinarum* and *Cu. lanceolata* showed a low-concentration promotion and high-concentration inhibition trend for dry weight of seedlings. Promotive effects on both fresh and dry weight were observed only at 0.25 g/mL concentration of aqueous extract of *L. formosana*. The aqueous extracts from *Pa. fortune*, *Ph. zhennan* and *Ch. axillaris* had significant inhibitory effects on dry weight of seedlings (**Figure 3**).

### 3.4 Effect of rhizosphere soil aqueous extracts on photosynthetic indices and chlorophyll fluorescence indexes of cuttings

**Figure 4:**
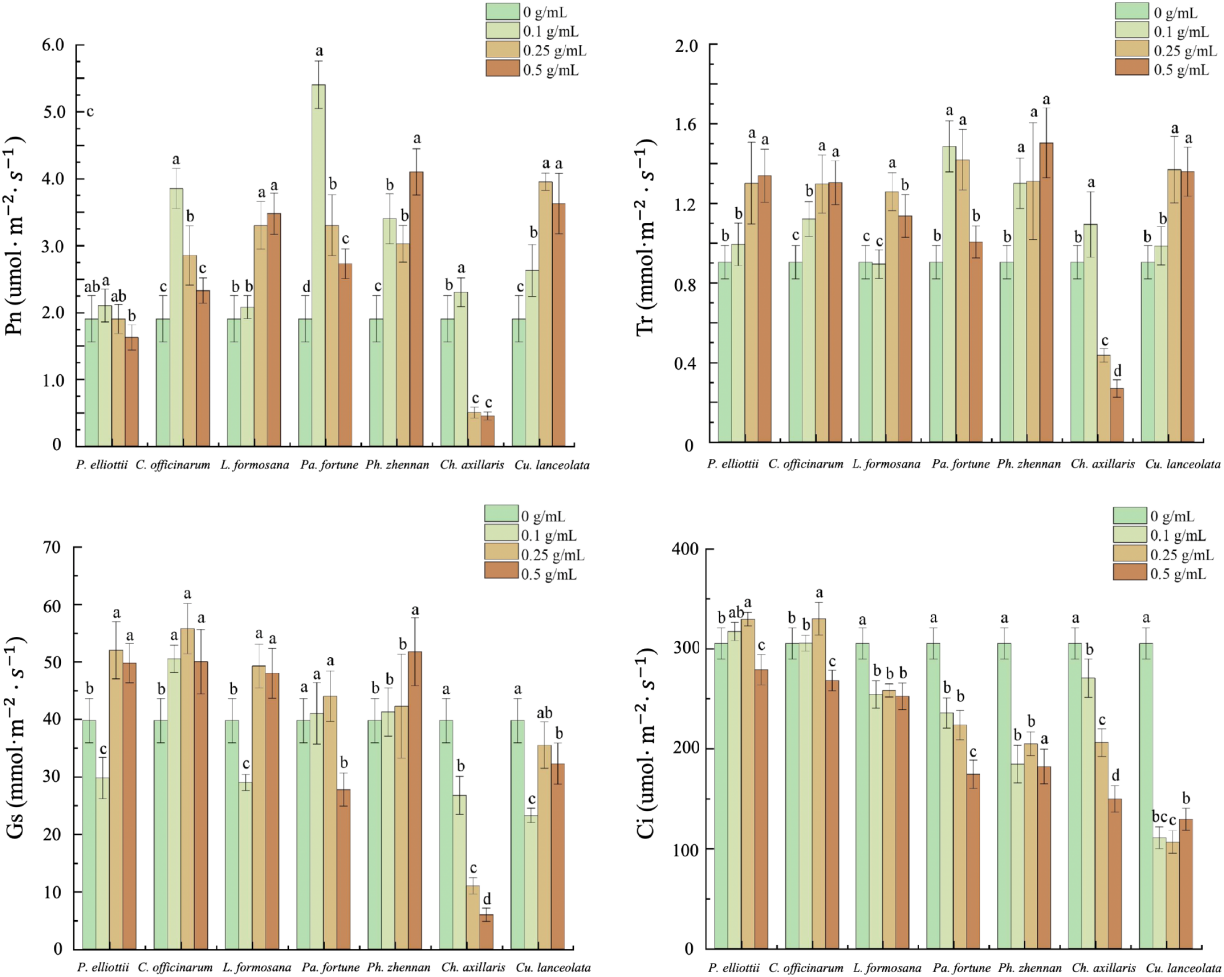
Effect of different concentrations of the rhizosphere soil aqueous extracts from seven trees on the Pn, Tr, Gs and Ci of *T. arguta*. Lowercase letters represent significant differences in each treatment group (p< 0.05).

Pn increased initially and then decreased with increasing concentration of aqueous extracts from *P. elliottii*, *C. officinarum, Pa. fortune* and *Ch. axillaris*, and increased with increasing concentration of aqueous extracts from *L. formosana* (**Figure 4**). The aqueous extracts from seven trees showed significant promoting effects on Tr, except for the high-concentration aqueous extracts of *Ch. axillaris*. The aqueous extracts from *Ch. axillaris* and *Cu. lanceolata* had significant inhibitory effects on Gs, but other five trees showed promoting effects. Only the aqueous extracts from *P. elliottii*, *C. officinarum* showed a low-concentration promotion and high-concentration inhibition trend for Ci, other five trees had significant inhibitory effects (**Figure 4**).

**Figure 5:**
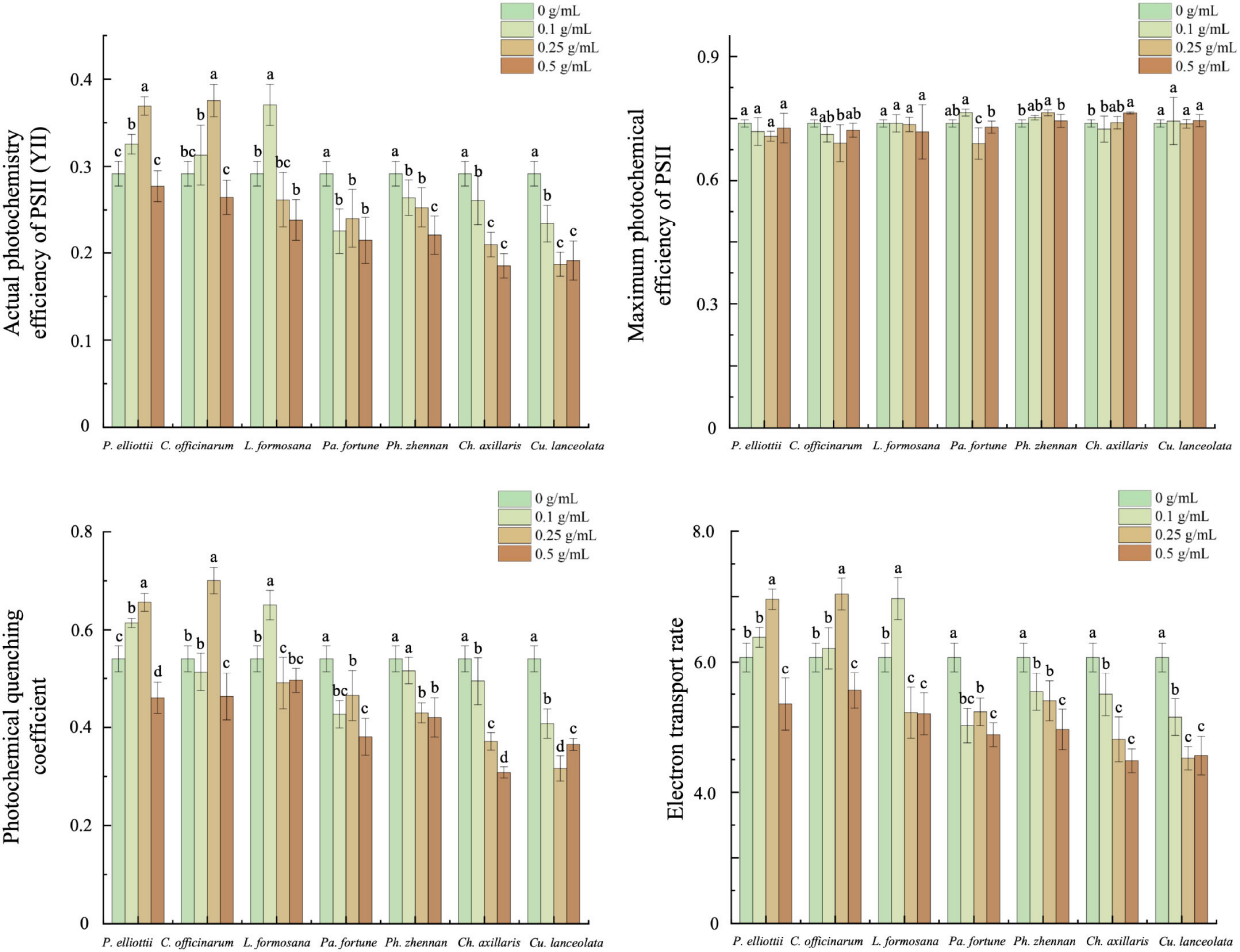
Effect of different concentrations of the rhizosphere soil aqueous extracts from seven trees on the PSII (YII), Maximum photochemical efficiency of PSII, qP and ETR of *T. arguta*. Lowercase letters represent significant differences in each treatment group (p< 0.05).

The aqueous extracts from *P. elliottii*, *C. officinarum* and *L. formosana* showed the maximum promotion at the concentration of 0.1 g/mL or 0.25 g/mL for actual photochemical efficiency of PSII (YII), qP and ETR, other four trees had significant inhibitory effects on these three indexes (**Figure 5**). The aqueous extracts from seven trees had no significant effects on maximum photochemical efficiency of PSII (**Figure 5**).

### 3.5 Effect of rhizosphere soil aqueous extracts on physiological and biochemical indices of cuttings

**Figure 6:**
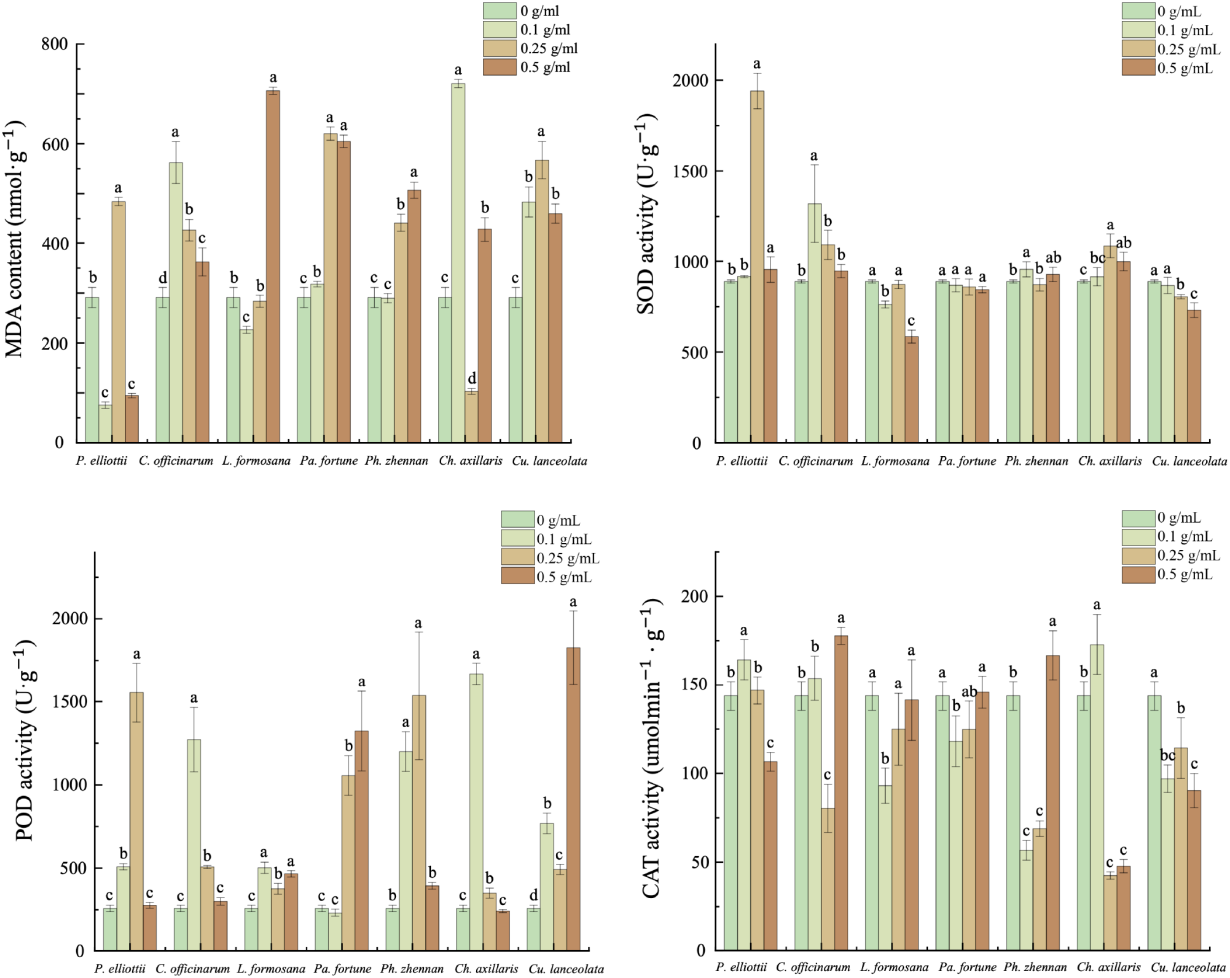
Effect of different concentrations of the rhizosphere soil aqueous extracts from seven trees on the MDA, SOD, POD and CAT of *T. arguta*. Lowercase letters represent significant differences in each treatment group (p< 0.05).

MDA content was significantly lower than that in control group at the concentration of 0.1 g/mL and 0.5 g/mL of the aqueous extracts from *P. elliottii*, but other six trees had certain degree of promoting effects on MDA (**Figure 6**). The aqueous extracts from *P. elliottii* showed a significant promoting effect on SOD activity at the concentration of 0.25 g/mL. The aqueous extracts from *C. officinarum* and *Ch. axillaris* also had significant promoting effects on SOD activity. The aqueous extracts from seven trees had promoting effects on POD activity, but the degree of effect varied from different concentrations. The aqueous extracts from *P. elliottii* and *Ch. axillaris* showed a low-concentration promotion and high-concentration inhibition trend for CAT activity, but *Ph. zhennan* showed a opposite trend. The aqueous extracts from *C. officinarum* had promoting effects on CAT activity at the concentration of 0.1 g/mL and 0.5 g/mL. The aqueous extracts from other three trees exhibited inhibitory effects on CAT activity (**Figure 6**).

### 3.6 Allelopathic effects of rhizosphere soil aqueous extracts

**Figure 7:**
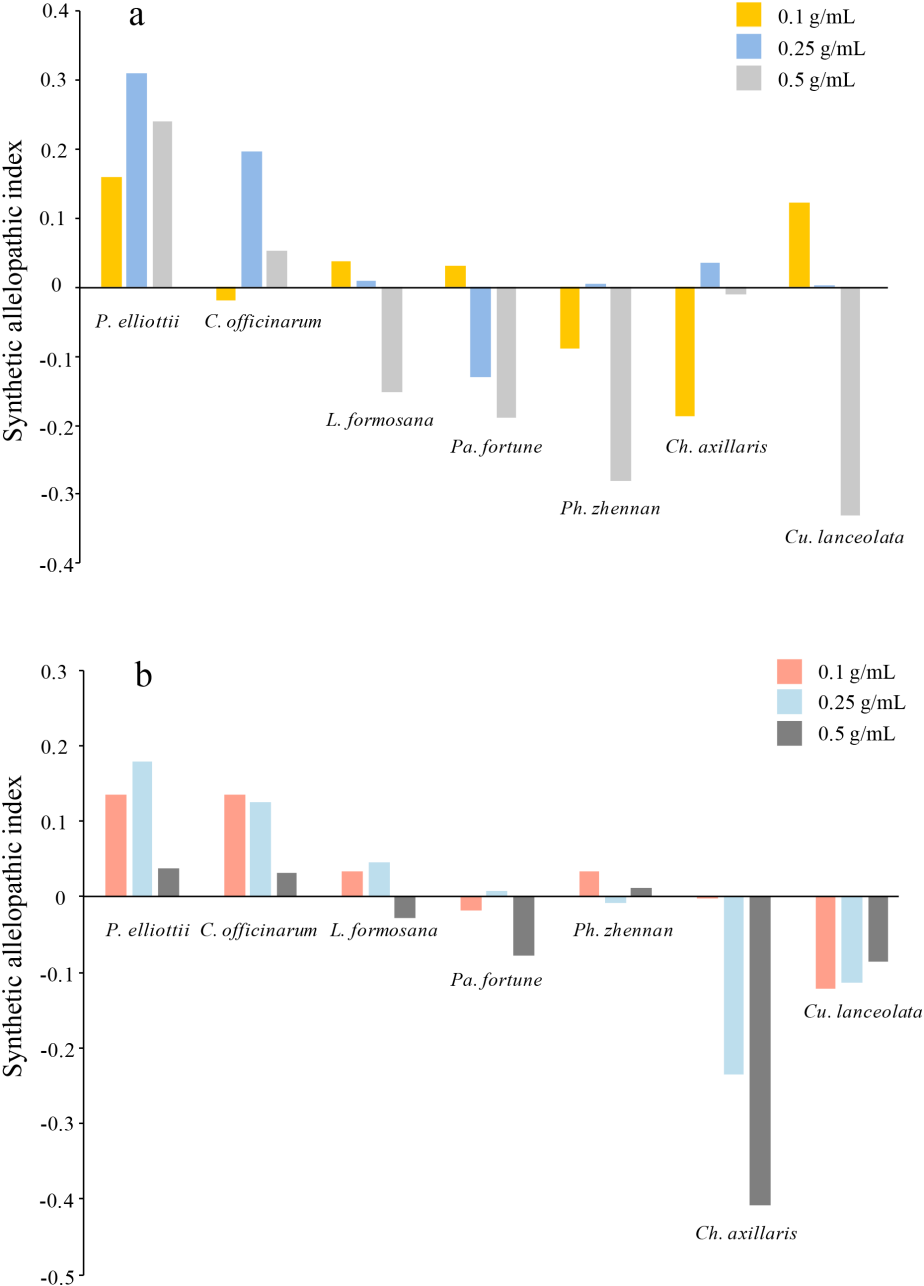
Allelopathic effects of rhizosphere soil aqueous extracts from seven trees on the growth of *T. arguta*. **(a)** Allelopathic effects of rhizosphere soil aqueous extracts on the seed germination and seedlings growth of *T. arguta*; **(b)** Allelopathic effects on cuttings growth of *T. arguta*.

Seven indicators (germination rate, germination potential, germination index, seedling height, longest root length, fresh weight and dry weight) were used to analyze the allelopathic effects of rhizosphere soil aqueous extracts on the seed germination and seedlings growth of *T. arguta*. The results showed that SE was affected by concentrations of aqueous extracts (**Figure 7a**). For example, SE value peaked at medium concentrations (0.25 g/mL) and decreased with increasing concentrations under the treatment of rhizosphere soil aqueous extracts from *P. elliottii, C. officinarum*. Low concentration had positive SE value, and high concentration had negative SE value for *L. formosana*, *Pa. fortune* and *Cu. lanceolata*. In addition, all the concentrations of *P. elliottii* had positive and maximum SE value. Twelve indicators (Pn, Tr, Gs, Ci, Actual photochemistry PSII (YII), Maximum photochemical efficiency of PSII, qP, ETR, MDA, SOD, POD, and CAT) were used to analyze the allelopathic effects for cuttings of *T. arguta*. The aqueous extracts from *P. elliottii* and *C. officinarum* had positive SE value for all concentrations, but *Ch. axillaris* and *Cu. lanceolata* had the negative SE value for all the treatments (**Figure 7b**). For *L. formosana*, *Pa. fortune* and *Ph. zhennan*, the promoting or inhibitory effects were depended on different concentrations (**Figure 7b**).

## 4 Discussion

### 4.1 Chemical components in rhizosphere soil aqueous extracts

The rhizosphere soil aqueous extracts from seven trees contained diverse chemical compounds, mainly including amides, alcohols, glycosides, phenols, acids, terpenes, ketones, esters and phenylpropanoids. Among these chemical compounds, 8α-Methacryloyloxy-13-ethoxyvernojalcanolide, a type of terpenoid, was found exclusively in the rhizosphere soil aqueous extract from *P. elliottii*. Terpenoids have been confirmed to exhibit allelopathic activity in other plant systems (Tholl, 2015; Yang et al., 2020; Jasim et al., 2023). Therefore, the presence of this specific terpenoid in *P. elliottii* suggests a potential-though unconfirmed-allelopathic role that could influence the growth of *T. arguta*. Further bioassay-guided fractionation and assays using purified compounds are needed to verify its functional role. Additionally, glycosides were uniquely identified in rhizosphere soil from *P. elliottii* and *C. officinarum*, with the predominant forms being 9’-β-D-xylopyranoside, Cinncassiol C1 19-glucoside and Delta-CEHC are known to function widely in plants as phytoalexins against biotic stress and antioxidant (Yang et al., 2018; Ali et al., 2022). It is plausible that the glycosides identified from *P. elliottii* and *C. officinarum* contribute to mitigating the degree of membrane lipid peroxidation in *T. arguta* cells, but direct experimental evidence is required to confirm this hypothesis. The Trolox was the most abundant compound in the rhizosphere soil of *C. officinarum*, which has been reported exhibiting superior peroxyl radical-scavenging activity in sodium dodecyl sulfate micelles and liposomes (Wu et al., 1990). Oleamide, linoleamide and obscuraminol E were consistently detected at high level in the rhizosphere soils of all seven trees. Previous studies have shown that Oleamide and linoleamide had significant antibacterial effects on *Pinellia ternata, Pseudostellaria radix* and *Morus alba* (Tang et al., 2004; Shi et al., 2024). These amides in the rhizosphere soil of seven tree species might inhibit harmful bacteria affecting the growth of *T. arguta*, however, this proposed mechanism remains speculative without targeted microbiological validation.

### 4.2 Effect of rhizosphere soil aqueous extracts on seed germination

Seed germination, as a critical process in plant life cycle that directly determines early competitiveness and population establishment (Barrero et al., 2014; Reed et al., 2022; Sikder et al., 2025). In this study, the rhizosphere soil aqueous extracts from seven trees exhibited significant effects on the seed germination of *T. arguta*. In general, low or medium concentration of rhizosphere soil aqueous extracts from seven tree species exhibited a considerable promoting effect on seed germination, whereas high concentrations weakened this promotional effect or even turned into inhibitory effect. Dual allelopathic effects (promotion and inhibition) on seed germination have been widely documented in previous studies (Cheng and Cheng, 2015). At moderate intensities, the allelochemicals in rhizosphere soil can impose mild stress on plant seeds. This stress increases cell membrane permeability, enhances internal respiration, elevates activity of the mitochondrial inner membrane, and accelerates metabolic rates, thereby improving seed vigor (Begum et al., 2024). However, when the allelopathic intensity exceeded the tolerance threshold of seeds, seed physiological deterioration may occur, leading to declines in multiple germination indices. Notably, the rhizosphere soil aqueous extracts from *P. elliottii* and *C. officinarum* showed significantly higher seed germination rate, germination potential and germination index than other tested species, indicating their superior promotional effects on the seed germination of *T. arguta*. Rhizosphere-specific glycosides identified in rhizosphere soil from *P. elliottii* and *C. officinarum* may play a pivotal role as determinant allelochemicals, though this requires experimental validation.

### 4.3 Effect of rhizosphere soil aqueous extracts on seedlings

The health of plant growth can be directly evaluated by measuring morphological indices such as root length, height, fresh weight and dry weight. As the primary interface for allelochemicals from rhizosphere soil, plant roots will exhibit the earliest physiological responses to allelopathic stress. Allelochemical exposure alters acquisition efficiency of water and nutrients of root system, subsequently affecting seedling growth. Compared with other six tree species, the rhizosphere soil aqueous extracts from *P. elliottii* not only exhibited the most significant promoting effect on the longest root length of *T. arguta*, but plant height, fresh weight and dry weight also had the greatest increment. Root traits such as root length strongly influence nutrients and water uptake efficiency (Bertin et al., 2003; Eissenstat and Volder, 2005; Holz et al., 2024), therefore, our results indicated that the root-determined allelochemical responsiveness may explain the growth of above-ground.

### 4.4 Effect of rhizosphere soil aqueous extracts on photosynthetic indices and chlorophyll fluorescence indexes of cuttings

Chloroplasts serve as the principal organelles for photon capture, energy transduction and photochemical transformation, holding critical importance for understanding the mechanisms of photosynthetic system. Photosynthetic parameters of plants are closely interrelated, clarifying their dynamic changes in these parameters helps to explore the allelopathic mechanisms from the perspective of photosynthetic physiology. Numerous studies have shown that root or leaf extracts can reduce Ci, Gs and Tr, thereby decreasing Pn (Yu et al., 2003; Zhu et al., 2009). In contrast, in this study, the rhizosphere soil aqueous extracts from seven trees had positive effects on Pn and Tr of *T. arguta* as a whole. On the one hand, for some species such as *C. officinarum* and *Ph. zhennan*, rhizosphere soil aqueous extracts significantly increased stomatal conductance. This improvement promoted leaf gas exchange (CO2 and water vapor), consequently boosting both photosynthetic and transpiration rates. On the other hand, allelochemicals can enhance plant metabolic efficiency. For instance, low or medium concentrations of rhizosphere soil aqueous extracts from *P. elliottii*, *C. officinarum* and *L. formosana* increased the PSII (YII), qP and ETR, which ultimately leads to higher overall photosynthetic efficiency.

### 4.5 Effect of rhizosphere soil aqueous extracts on physiological and biochemical indices of cuttings

MDA is a product of lipid peroxidation in cell membranes, commonly used for assessing the degree of oxidative stress and stress resistance in plant cells, elevated MDA content indicates greater oxidative damage to plant cell membranes. Environmental stress induces excessive accumulation of reactive oxygen species in plant cells, which exceeds the clearance capacity of endogenous antioxidant systems and ultimately causes oxidative damage. Correspondingly, three major antioxidant enzymes (SOD, POD and CAT) will upregulate in plants (Ding et al., 2007; Rout et al., 2013). SOD can eliminate metabolically derived free radicals and other deleterious compounds, and SOD activity level directly correlates with plant viability and mortality (Stephenie et al., 2020). Moreover, POD and CAT detoxify H_2_O_2_ produced by SOD-mediated dismutation, thereby protecting cells from oxidative damage. Our results showed that most of the treatments increased MDA content, accompanied by synchronous changes in the activities of SOD, POD, and CAT. For example, MDA content increased significantly under the treatment of rhizosphere soil aqueous extract from *P. elliottii* at the concentration of 0.25 g/mL, along with a dramatic enhancement in SOD and POD activities. In summary, treatments with rhizosphere soil aqueous extracts impacted the antioxidant system of *T. arguta*. Three protective enzymes performed specific but complementary phusiological functions and acted synergistically to sustain cellular redox homeostasis in *T. arguta*.

### 4.6 Allelopathic effects of rhizosphere soil aqueous extracts

The synthetic allelopathic index is a vital indicator for evaluating the intensity of plant allelopathic effects. Much research has shown that allelopathy plays a dual role, potentially both promoting and inhibiting plant growth (Song, 2012; Kong et al., 2013; Yeo et al., 2014; Mohsin et al., 2016; Christoph et al., 2017). Consistent with previous findings, our study revealed that the allelopathic effects of rhizosphere soil aqueous extracts on the growth of *T. arguta* were facilitative at low concentrations but inhibitory at high concentrations (Wang et al., 2019; Li et al., 2021b). In addition, pot experiments on cuttings demonstrated that allelopathy affects plant growth by influencing photosynthesis and physiological and biochemical processes. Certain allelochemicals can promote the growth of recipient plants by exerting hormone-like effects or by modulating the composition and concentration of endogenous hormones within them (Ren et al., 2018). In this study, we found that the rhizosphere soil aqueous extract of *P. elliottii* exhibited the most pronounced promoting effect on the growth of *T. arguta*, and specific terpenoid or glycosides in rhizosphere soil aqueous extract from *P. elliottii* are presumed to be the core allelochemicals responsible for this dominant facilitative effect. However, this interpretation remains speculative without experimental validation. Given that the allelopathic effects on *T. arguta* vary from different tree species and are concentration-dependent, both tree species and concentration dependence should be comprehensively considered in the understory planting.

## 5 Conclusions

**Figure 8:**
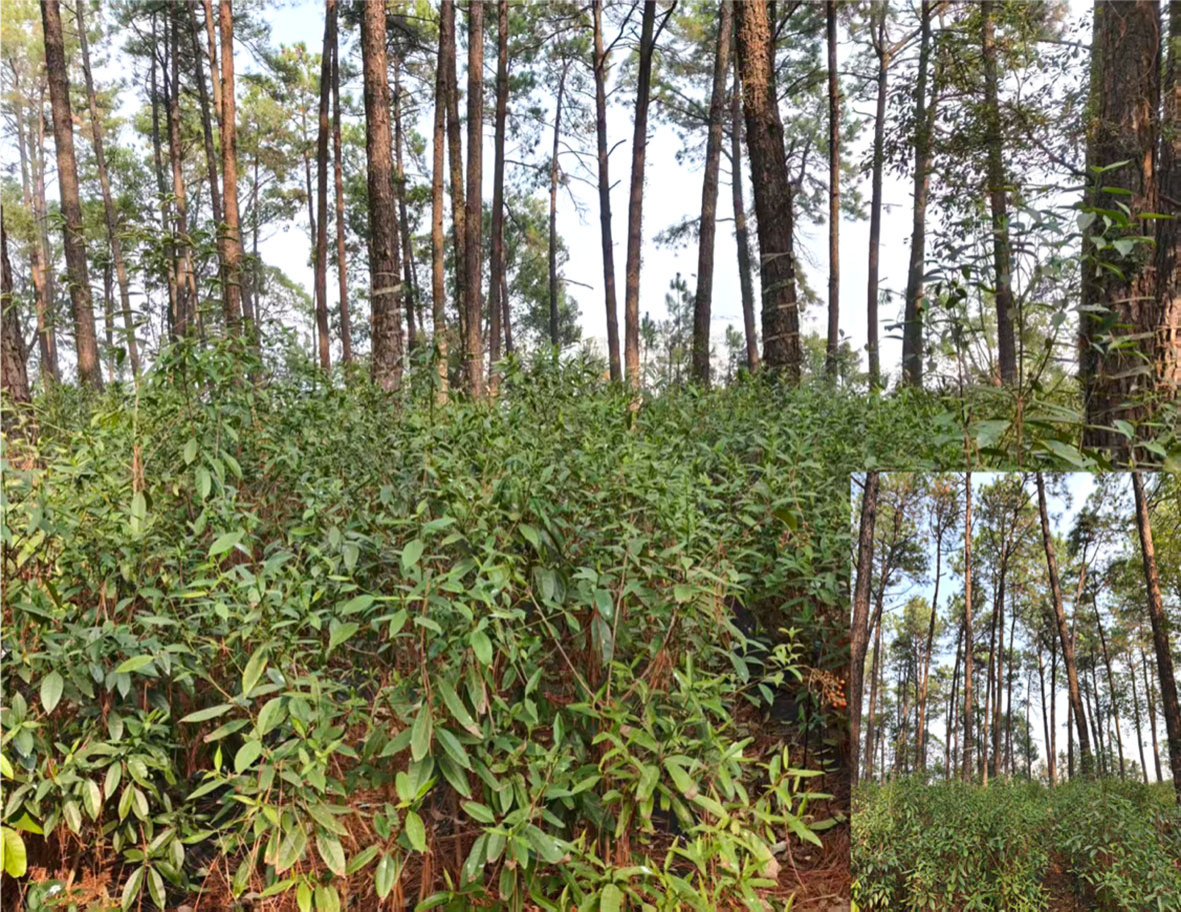
Field validation of the recommended agroforestry system. The photograph shows the operational *P. elliottii* - *T. arguta* understory cultivation model established at the Jiangxi Academy of Forestry, Nanchang. This model exemplifies the efficient utilization of forest understory space for the sustainable production of this high-value medicinal plant.

The development of sustainable cultivation systems for high-value medicinal plant like *T. arguta* requires a detailed understanding of ecological interactions between plants. This study provides novel evidence that allelopathic interactions between *T. arguta* and its companion trees are concentration-dependent, following a low-concentration promotion and high-concentration inhibition pattern. Among the seven tree species evaluated, *P. elliottii* exhibited the most consistently promotive effects across multiple growth stages and physiological parameters. This study provides a theoretical basis for optimizing species selection in understory agroforestry and suggest an integrated *P. elliottii* - *T. arguta* understory planting system. The practical viability of this approach is further supported by field validation, where *P. elliottii* - *T. arguta* planting model achieve over 95% survival rates and vigorous growth (**Figure 8**), demonstrating its potential for industrial cultivation.

### 5.1 Study limitations and future directions

This study has some limitations: the small sample size limits statistical power and makes the findings preliminary; the limited concentration gradient precluded determining maximum inhibitory concentrations for some species such as *P. elliottii* and *C. officinarum*; and the specific allelochemicals remain unidentified. Future work should address these gaps through field trials measuring yield and bioactive compounds and isolation of key allelochemicals.

## Data Availability

The original contributions presented in the study are included in the article/**Supplementary Material**. Further inquiries can be directed to the corresponding authors.
