# Supplementary material for: Developing an efficient intercrop system for understory medicinal plant cultivation: allelopathy- based screening of tree species for Turpinia arguta
Source: Front Plant Sci. 2026 Jul 3;17:1878126. doi: 10.3389/fpls.2026.1878126 (PMC13377982; doi:10.3389/fpls.2026.1878126)
Supplement: Supplementary file 1 [file Table1.docx]

| Chemical name | | Molecular formula | | Relative content | |
| --- | --- | --- | --- | --- | --- |
| Oleamide | | C_18_H_35_NO | | 12.86 | |
| 8α-Methacryloyloxy-13-ethoxyvernojalcanolide | | C_25_H_34_O_11_ | | 11.62 | |
| Linoleamide | | C_18_H_33_NO | | 11.58 | |
| 5-O-Ethylcleroindicin D | | C_10_H_16_O_4_ | | 9.30 | |
| Obscuraminol E | | C_16_H_33_NO | | 8.37 | |
| (7R*,8R*)-3-Methoxy-3',4,7,9,9'-pentahydroxy-8,4'-oxyneolignan | | C_19_H_24_O_7_ | | 5.31 | |
| Apocynol A | | C_13_H_20_O_3_ | | 4.41 | |
| Juncuenin D | | C_18_H_18_O_3_ | | 3.58 | |
| (E)-2-Octen-1-ol | | C_8_H_16_O | | 3.42 | |
| 2-(4-oxo-6,7,8,9-tetrahydropyrimido[4,5-b] quinolin-3-yl)-N-(2-phenylethyl) acetamide | | C_21_H_22_N_4_O_2_ | | 2.67 | |
| 5-Aminopentanoic acid | | C_5_H_11_NO_2_ | | 2.62 | |
| 9'-β-D-xylopyranoside | | C_25_H_32_O_10_ | | 2.37 | |
| Cinncassiol C1 19-glucoside | | C_26_H_38_O_12_ | | 2.28 | |
| p-Salicylic acid | | C_7_H_6_O_3_ | | 2.02 | |
| Gentisic acid | | C_7_H_6_O_4_ | | 1.99 | |
| 2-(5,8-Tetradecadienyl) cyclobutanone | | C_18_H_30_O | | 1.97 | |
| 5-Megastigm-7-ene-3,4,6,9-tetrol | | C_13_H_24_O_4_ | | 1.90 | |
| Tinospinoside C | | C_27_H_36_O_12_ | | 1.80 | |
| Vanillic acid | | C_8_H_8_O_4_ | | 1.63 | |
| (3R,8E)-3-Hydroxy-5,8-megastigmadien-7-one | | C_13_H_20_O_2_ | | 1.55 | |
| 16-Methylheptadecanoic acid | | C_18_H_36_O_2_ | | 1.43 | |
| Phaseolus e | | C_25_H_34_O_12_ | | 1.36 | |
| Cumic alcohol | | C_10_H_14_O | | 1.34 | |
| Retrocalamin | | C_24_H_30_O_9_ | | 1.32 | |
| Valine | | C_5_H_11_NO_2_ | | 1.31 | |

Supplementary material

Table S1 LC-MS analysis of rhizosphere soil aqueous extracts of *Pinus elliottii*

Table S2 LC-MS analysis of rhizosphere soil aqueous extracts of *Camphora officinarum*

| Chemical name | Molecular formula | Relative content |
| --- | --- | --- |
| Trolox | C_14_H_18_O_4_ | 16.28 |
| Stearic acid amide | C_18_H_37_NO | 10.47 |
| Dihydro-3-coumaric acid | C_9_H_10_O_3_ | 9.48 |
| Orthothymotinic acid | C_11_H_14_O_3_ | 6.16 |
| Sebacic acid | C_10_H_18_O_4_ | 6.02 |
| Oleamide | C_18_H_35_NO | 5.67 |
| Boscialin | C_13_H_22_O_3_ | 4.58 |
| p-Salicylic acid | C_7_H_6_O_3_ | 3.90 |
| Pipecolinic acid | C_6_H_11_NO_2_ | 3.61 |
| Linoleamide | C_18_H_33_NO | 3.54 |
| Obscuraminol E | C_16_H_33_NO | 3.47 |
| 3-hydroxy-3-[4-hydroxy-3-(3-methylbut-2-en-1-yl) phenyl] propanoic acid | C_14_H_18_O_4_ | 3.23 |
| cis-p-Menthane-1,7,8-triol | C_10_H_20_O_3_ | 3.03 |
| Delta-CEHC | C_14_H_18_O_4_ | 2.93 |
| trans-p-Menth-2-ene-1,4-diol | C_10_H_18_O_2_ |  |
| 5-Megastigm-7-ene-3,4,6,9-tetrol | C_13_H_24_O_4_ | 2.02 |
| Plucheoside B aglycone | C_13_H_22_O_3_ | 1.79 |
| p-Mentha-1,3,8-triene | C_10_H_14_ | 1.72 |
| Aegelinol | C_14_H_14_O_4_ | 1.70 |
| 4-Hydroxybenzoic acid | C_7_H_6_O_3_ | 1.52 |
| Methyl jasmonate | C_13_H_20_O_3_ | 1.48 |
| 3-Cresotinic acid | C_8_H_8_O_3_ | 1.47 |
| Polygalic acid | C_29_H_44_O_6_ | 1.36 |
| Ectoine | C_6_H_10_N_2_O_2_ | 1.20 |
| 2-Ethylglutaric acid | C_7_H_12_O_4_ | 1.18 |

Table S3 LC-MS analysis of rhizosphere soil aqueous extracts of *Liquidambar formosana*

| Chemical name | Molecular formula | Relative content |
| --- | --- | --- |
| Oleamide | C_18_H_35_NO | 24.99 |
| Linoleamide | C_18_H_33_NO | 23.94 |
| Obscuraminol E | C_16_H_33_NO | 16.27 |
| (E)-2-Octen-1-ol | C_8_H_16_O | 5.45 |
| Stearic acid amide | C_18_H_37_NO | 4.36 |
| 2-(5,8-Tetradecadienyl) cyclobutanone | C_18_H_30_O | 4.08 |
| Cinobufagin | C_26_H_34_O_6_ | 2.31 |
| 16-Methylheptadecanoic acid | C_18_H_36_O_2_ | 2.16 |
| Ascorbyl palmitate | C_22_H_38_O_7_ | 2.16 |
| 5-Aminopentanoic acid | C_5_H_11_NO_2_ | 1.97 |
| Secoxyloganin | C_17_H_24_O_11_ | 1.83 |
| 1-Nonanol | C_9_H_20_O | 1.80 |
| Dendryphiellic acid A | C_9_H_14_O_2_ | 0.91 |
| 2-(5-Tetradecenyl) cyclobutanone | C_18_H_32_O | 0.90 |
| 2-Undecen-1-ol | C_11_H_22_O | 0.88 |
| (7R*,8R*)-3-Methoxy-3',4,7,9,9'-pentahydroxy-8,4'-oxyneolignan | C_19_H_24_O_7_ | 0.73 |
| 8-Amino-7-oxononanoic acid | C_9_H_17_NO_3_ | 0.72 |
| Genipic acid | C_9_H_12_O_4_ | 0.67 |
| Carnitine | C_7_H_15_NO_3_ | 0.63 |
| Lactoferrin | C_27_H_47_N_7_O_6_ | 0.62 |
| Sangivamycin | C_12_H_15_N_5_O_5_ | 0.59 |
| 4-Hydroxy-6-methyl-3-(1-oxobutyl)-2H-pyran-2-one | C_10_H_12_O_4_ | 0.55 |
| Mangiferic acid | C_18_H_32_O_2_ | 0.50 |
| N, N-Dimethyldodecylamine N-oxide | C_14_H_31_NO | 0.50 |
| Sebacic acid | C_10_H_18_O_4_ | 0.47 |

Table S4 LC-MS analysis of rhizosphere soil aqueous extracts of *Paulownia fortunei*

| Chemical name | Molecular formula | Relative content |
| --- | --- | --- |
| Oleamide | C_18_H_35_NO | 28.53 |
| Linoleamide | C_18_H_33_NO | 26.64 |
| Obscuraminol E | C_16_H_33_NO | 17.83 |
| 2-(5,8-Tetradecadienyl) cyclobutanone | C_18_H_30_O | 4.44 |
| (E)-2-Octen-1-ol | C_8_H_16_O | 4.31 |
| Cinobufagin | C_26_H_34_O_6_ | 2.71 |
| 1-Nonanol | C_9_H_20_O | 2.08 |
| 16-Methylheptadecanoic acid | C_18_H_36_O_2_ | 2.02 |
| 2-Undecen-1-ol | C_11_H_22_O | 1.60 |
| Stearic acid amide | C_18_H_37_NO | 1.27 |
| 5-Aminopentanoic acid | C_5_H_11_NO_2_ | 1.09 |
| 2-(5-Tetradecenyl) cyclobutanone | C_18_H_32_O | 1.07 |
| Mesylate | CH_4_O_3_S | 0.84 |
| Carnitine | C_7_H_15_NO_3_ | 0.75 |
| Lactoferrin | C_27_H_47_N_7_O_6_ | 0.60 |
| 1-Monopalmitin | C_19_H_38_O_4_ | 0.54 |
| Mulberrofuran M | C_9_H_10_ClNO_2_ | 0.49 |
| N, N-Dimethyldodecylamine N-oxide | C_14_H_31_NO | 0.49 |
| alpha-Ecdysone | C_27_H_44_O_6_ | 0.48 |
| 6-Gingerol | C_17_H_26_O_4_ | 0.45 |
| Mangiferic acid | C_18_H_32_O_2_ | 0.39 |
| Valine | C_5_H_11_NO_2_ | 0.38 |
| D-phenylalanine | C_9_H_11_NO_2_ | 0.34 |
| 1,20-Eicosanediol | C_20_H_42_O_2_ | 0.34 |
| 3-(4-hydroxy-3-methoxyphenyl) oxirane-2-carboxylic acid | C_10_H_10_O_5_ | 0.33 |

Table S5 LC-MS analysis of rhizosphere soil aqueous extracts of *Phoebe zhennan*

| Chemical name | Molecular formula | Relative content |
| --- | --- | --- |
| Oleamide | C_18_H_35_NO | 17.49 |
| Stearic acid amide | C_18_H_37_NO | 16.93 |
| Linoleamide | C_18_H_33_NO | 14.89 |
| Obscuraminol E | C_16_H_33_NO | 10.89 |
| C (10S,11S)-Pterosin C | C_14_H_18_O_3_ | 4.90 |
| (+)-Galeon | C_20_H_22_O_4_ | 4.56 |
| (E)-2-Octen-1-ol | C_8_H_16_O | 4.06 |
| (+)-Eudesmin | C_22_H_26_O_6_ | 3.22 |
| Norisoboldine | C_18_H_19_NO_4_ | 3.04 |
| 2-(5,8-Tetradecadienyl) cyclobutanone | C_18_H_30_O | 2.52 |
| 5-hydroxy-8-(2-hydroxypropan-2-yl)-4-propyl-2H,8H,9H-furo[2,3-h] chromen-2-one | C_17_H_20_O_5_ | 1.70 |
| (E)-N-[4-(7-methylimidazo[1,2-a] pyrimidin-2-yl) phenyl]-3-phenylprop-2-enamide | C_22_H_18_N_4_O | 1.70 |
| Cinobufagin | C_26_H_34_O_6_ | 1.65 |
| 16-Methylheptadecanoic acid | C_18_H_36_O_2_ | 1.58 |
| Lucidone B | C_24_H_32_O_5_ | 1.51 |
| 1-Nonanol | C_9_H_20_O | 1.44 |
| Kanzonol N | C_22_H_24_O_6_ | 1.37 |
| Reticuline | C_19_H_23_NO_4_ | 1.12 |
| PGF2a ethanolamide | C_22_H_39_NO_5_ | 0.91 |
| trans-Norpterosin C | C_13_H_16_O_3_ | 0.86 |
| 3-(7-hydroxy-5-methoxy-2,2-dimethyl-2H-chromen-6-yl)-1-phenylprop-2-en-1-one | C_21_H_20_O_4_ | 0.84 |
| Mesylate | CH_4_O_3_S | 0.76 |
| 2-Undecen-1-ol | C_11_H_22_O | 0.72 |
| Lariciresinol acetate | C_22_H_26_O_7_ | 0.69 |
| Carnitine | C_7_H_15_NO_3_ | 0.66 |

Table S6 LC-MS analysis of rhizosphere soil aqueous extracts of *Choerospondias axillaris*

| Chemical name | Molecular  formula | Relative content |
| --- | --- | --- |
| Stearic acid amide | C_18_H_37_NO | 22.33 |
| Oleamide | C_18_H_35_NO | 20.08 |
| Linoleamide | C_18_H_33_NO | 17.92 |
| Obscuraminol E | C_16_H_33_NO | 13.18 |
| (E)-2-Octen-1-ol | C_8_H_16_O | 4.29 |
| 2-(5,8-Tetradecadienyl) cyclobutanone | C_18_H_30_O | 3.05 |
| Cinobufagin | C_26_H_34_O_6_ | 2.97 |
| 5-Aminopentanoic acid | C_5_H_11_NO_2_ | 2.28 |
| 16-Methylheptadecanoic acid | C_18_H_36_O_2_ | 1.96 |
| 1-Nonanol | C_9_H_20_O | 1.80 |
| Carnitine | C_7_H_15_NO_3_ | 1.30 |
| 2-Undecen-1-ol | C_11_H_22_O | 1.19 |
| Sebacic acid | C_10_H_18_O_4_ | 0.93 |
| Mesylate | CH_4_O_3_S | 0.91 |
| Nalpha-Acetyl-L-lysine | C_8_H_16_N_2_O_3_ | 0.81 |
| 2-(5-Tetradecenyl) cyclobutanone | C_18_H_32_O | 0.73 |
| Lactoferrin | C_27_H_47_N_7_O_6_ | 0.59 |
| N, N-Dimethyldodecylamine N-oxide | C_14_H_31_NO | 0.54 |
| 4-Guanidinobutanoic acid | C_5_H_11_N_3_O_2_ | 0.52 |
| p-Salicylic acid | C_7_H_6_O_3_ | 0.52 |
| 2-Ethylglutaric acid | C_7_H_12_O_4_ | 0.51 |
| Pipecolinic acid | C_6_H_11_NO_2_ | 0.43 |
| 3-Cresotinic acid | C_8_H_8_O_3_ | 0.39 |
| Mangiferic acid | C_18_H_32_O_2_ | 0.39 |
| 1, 20-Eicosanediol | C_20_H_42_O_2_ | 0.36 |

Table S7 LC-MS analysis of rhizosphere soil aqueous extracts of *Cunninghamia lanceolate*

| Chemical name | Molecular formula | Relative content |
| --- | --- | --- |
| Oleamide | C_18_H_35_NO | 18.84 |
| Stearic acid amide | C_18_H_37_NO | 16.11 |
| Linoleamide | C_18_H_33_NO | 15.84 |
| Obscuraminol E | C_16_H_33_NO | 12.87 |
| 5-Aminopentanoic acid | C_5_H_11_NO_2_ | 7.53 |
| (E)-2-Octen-1-ol | C_8_H_16_O | 4.70 |
| 2-(5,8-Tetradecadienyl) cyclobutanone | C_18_H_30_O | 2.71 |
| Cinobufagin | C_26_H_34_O_6_ | 2.56 |
| p-Salicylic acid | C_7_H_6_O_3_ | 2.15 |
| 16-Methylheptadecanoic acid | C_18_H_36_O_2_ | 2.02 |
| 1-Nonanol | C_9_H_20_O | 1.89 |
| Valine | C_5_H_11_NO_2_ | 1.79 |
| 2-Undecen-1-ol | C_11_H_22_O | 1.39 |
| Solanapyrone B | C_18_H_24_O_4_ | 1.25 |
| Panaquinquecol 6 | C_19_H_26_O_4_ | 1.17 |
| Cholinesulfuric acid | C_5_H_13_NO_4_S | 0.92 |
| D-phenylalanine | C_9_H_11_NO_2_ | 0.88 |
| Carnitine | C_7_H_15_NO_3_ | 0.75 |
| Gentisic acid | C_7_H_6_O_4_ | 0.73 |
| Vanillic acid | C_8_H_8_O_4_ | 0.70 |
| 2-(5-Tetradecenyl) cyclobutanone | C_18_H_32_O | 0.69 |
| Lactoferrin | C_27_H_47_N_7_O_6_ | 0.65 |
| Sangivamycin | C_12_H_15_N_5_O_5_ | 0.64 |
| artemisinin | C_15_H_22_O_5_ | 0.63 |
| 2-Ethylglutaric acid | C_7_H_12_O_4_ | 0.60 |
